# Turning Spent Coffee Grounds into Sustainable Precursors for the Fabrication of Carbon Dots

**DOI:** 10.3390/nano10061209

**Published:** 2020-06-21

**Authors:** Diana M. A. Crista, Abderrahim El Mragui, Manuel Algarra, Joaquim C. G. Esteves da Silva, Rafael Luque, Luís Pinto da Silva

**Affiliations:** 1 Chemistry Research Unit (CIQUP), Faculty of Sciences of University of Porto, R. Campo Alegre 697, 4169-007 Porto, Portugal; dianacrista4@gmail.com (D.M.A.C.); a.elmragui@edu.umi.ac.ma (A.E.M.); jcsilva@fc.up.pt (J.C.G.E.d.S.); 2 Department of Inorganic Chemistry, Faculty of Science, University of Málaga, Campus de Teatino s/n, 29071 Málaga, Spain; 3 LACOMEPHI, GreenUPorto, Department of Geosciences, Environment and Territorial Planning, Faculty of Sciences of University of Porto, R. Campo Alegre 697, 4169-007 Porto, Portugal; 4 Departamento de Química Orgánica, Universidad de Córdoba, Edif. Marie Curie, Ctra N-IVA Km 396, E-14014 Córdoba, Spain; 5 Center for Molecular Design and Synthesis of Innovative compounds for Medicine, Peoples Friendship University of Russia (RUDN University), 6 Miklukho Maklaya Street, 117198 Moscow, Russia

**Keywords:** carbon dots, nanotechnology, fluorescence, spent coffee grounds, life cycle assessment, sensing

## Abstract

Spent coffee grounds (SCGs) are known for containing many organic compounds of interest, including carbohydrates, lipids, phenolic compounds and proteins. Therefore, we investigated them as a potential source to obtain carbon dots (CDs) via a nanotechnology approach. Herein, a comparison was performed between CDs produced by SCGs and classic precursors (e.g., citric acid and urea). The SCG-based CDs were obtained via the one-pot and solvent-free carbonization of solid samples, generating nanosized particles (2.1–3.9 nm). These nanoparticles exhibited a blue fluorescence with moderate quantum yields (2.9–5.8%) and an excitation-dependent emission characteristic of carbon dots. SCG-based CDs showed potential as environmentally relevant fluorescent probes for Fe^3+^ in water. More importantly, life cycle assessment studies validated the production of CDs from SCG samples as a more environmentally sustainable route, as compared to those using classic reported precursors, when considering either a weight- or a function-based functional unit.

## 1. Introduction

Carbon dots (CDs) are fluorescent carbon-based nanomaterials with a near-spherical shape and are typically sized below 10 nm [1,2]. CDs possess remarkable properties, including high photoluminescence, biocompatibility, high photostability and chemical stability, good water solubility and simplicity of functionalization [3,4,5,6,7]. Thus, it is not surprising that CDs have emerged as a suitable replacement for more traditional fluorophores, being used in several fields, such as the fabrication of light emission devices, bioimaging, sensing, drug delivery and biomedicine [2,8,9,10,11].

Another very attractive feature of CDs is that they can be fabricated from a large variety of precursors without expensive and/or toxic materials or sophisticated equipment [12,13,14,15]. In fact, their general synthetic route is based on the simple thermal or microwave-assisted treatment of organic molecules as a carbon source (generally citric acid), together with other organic molecules as a nitrogen source (e.g., urea as a common nitrogen source), both in aqueous solution or in powder form [16,17,18]. These approaches make CDs a sustainable and non-toxic alternative to metal-based quantum dots. However, most available organic molecules are still expensive, and their use and/or synthesis can pose significant challenges to the environment and to human health [19]. It has become quite desirable to use biomass waste as alternative precursors in the synthesis of CDs [20], given that biomass waste material is ubiquitous, nontoxic, cheap and renewable [21]. Furthermore, the use of such biomass would allow the valorization of waste material of other industries, a desirable outcome toward the establishment of a circular economy.

One of the biomass waste materials of increasing importance is spent coffee grounds (SCGs), which are the residues obtained from treatment of coffee powder with hot water or steam during the brewing process [22]. In fact, coffee consumption has increased significantly in recent years, with a worldwide consumption of over 9.5 million tons in 2017. Among the different ways of consuming coffee, one of the most popular ways is by using disposable espresso capsules, being a method adopted in homes, offices and cafeterias [23]. This significant increase in coffee consumption has consequently led to an increase in waste products, including used coffee capsules containing coffee grounds. In fact, their increasing numbers has forced companies to create various recycling programs. For example, Nespresso created a worldwide collection system consisting of 14,000 collection points and over 88,000 United Parcel Service (UPS) drop off points in the USA. Residues from espresso coffee consumption are also considered to be the main waste generated by cafeterias widespread all over the world. In simple terms, used coffee capsules are composed by the capsule itself and by SCGs [22], with most of a used capsule’s weight corresponding to SCGs. Thus, SCGs are becoming a quite problematic waste product. Interestingly, SCGs have been found to contain many organic compounds of interest, such as carbohydrates, lipids, phenolic compounds and proteins [24]. Thus, SCG waste has significant potential for valorization.

Given this, it is not surprising that several researchers have already used SCGs as precursors for the fabrication of CDs. Chang and co-workers pioneered the use of SCGs in the fabrication of CDs, by performing the one-pot thermal carbonization of the samples and obtaining nanoparticles with a size of 5 ± 2 nm and a fluorescent quantum yield of 3.8% [25]. These CDs were then used in cell imaging and surface-assisted laser desorption/ionization mass spectrometry (SALDI-MS) of angiotensin I and insulin. CDs with similar properties (a size of 4.4 nm and a quantum yield of 5.5%) were also tested for cells and fish imaging [26]. SCG-based CDs were also applied for the sensing of heavy metals, [27] after synthesis via the hydrothermal treatment of SCGs in the presence of hydrazine and subsequent functionalization with poly(ethylene imine), from which resulted nanoparticles with average sizes between 1.88 ± 0.72 nm and 2.67 ± 0.81 nm. Finally, Li and co-workers have prepared CDs from the treatment of coffee bean shells with an aqueous sodium hydroxide solution, followed by precipitation with hydrochloric acid. This resulted in the formation of CDs with diameters ranging from 1 to 5 nm, which were later used for banana storage (based on their antioxidation ability), and the imaging of cellular nuclei and in vivo bioimaging [28].

These results demonstrated that SCGs can indeed be used in the fabrication of CDs with relevant practical applications (bioimaging and sensing), which indicated that SCGs could provide a more environmentally sustainable route for the synthesis of CDs. However, these studies failed to assess some significant aspects. Namely, they did not investigate the environmental impacts associated with the synthesis of CDs from SCGs. They also did not compare the properties of the SCG-based CDs with those of CDs obtained from standard precursors (such as citric acid and urea). Thus, while the claim was made, there is not enough information to determine if the use of SCGs as precursors can indeed lead to a more environmentally sustainable fabrication of CDs, when in comparison with the use of standard precursors. This can be particularly problematic if we consider that, while the use of standard precursors might lead to a less sustainable synthesis route (when considering only the amount of CDs), they also might lead to improved properties that could justify a more resource-intensive synthesis.

It should be pointed out that most studies found in the literature regarding CDs are focused on the development of new individual nanoparticles with novel applications at the laboratory scale. However, and despite the hundreds to thousands of such papers, the translation of this vast research to industrial/commercial applications is non-existent. This is due to the fact that, while each new study uses different synthesis protocols and/or precursors, leading to almost unrepeatable nanoparticles, there are only limited data on trends that may allow us to predict the structure/surface functionalization and photoluminescence of CDs. This makes the reproduction of existent results difficult and prevents the rational development of new CDs, leaving the research community to serendipitous discoveries, given that there has been an insufficient effort to develop a well-defined and reproducible scaffold structure for CDs, which researchers can use to improve and modify in a rational and incremental way toward new applications. 

Herein, we have compared the fabrication of CDs prepared from different SCG samples and from standard precursors (citric acid and urea), [16,29,30] via a one-pot and solvent-free carbonization method [21]. The objective of this comparison is to determine if the use of SCGs as precursors really leads to a more environmentally sustainable synthesis route than the use of standard precursors, even when the functional properties of all CDs are considered (such as synthesis yield and fluorescent quantum yield). Our work also aims to determine if different SCGs can be used as a family of precursors that can generate reproducible and predictable CDs, with identical structure/surface functionalization and photoluminescence, and in this way lead to the formation of common and dependable CD scaffolds.

To this end, the resulting CDs were characterized by atomic force microscopy (AFM), dynamic light scattering (DLS) and X-ray photoelectron (XPS), UV–Vis and fluorescence spectroscopy. A comparative life cycle assessment (LCA) study was also performed for all CDs, to determine which is the most environmentally sustainable fabrication method for fluorescent CDs.

## 2. Materials and Methods 

### 2.1. Materials 

Three different SCG samples were obtained from three different used espresso capsules, each one corresponding to different Portuguese coffee brands: Chave D’Ouro^®^ descafeínado (decaffeinated) blend, Nicola^®^ Rossio (intense) blend, and Chave D’Ouro^®^ Prestígio (Prestige) blend. Citric acid and urea were purchased from Sigma-Aldrich (St. Louis, MI, USA).

### 2.2. Fabrication of CDs

CDs were fabricated via a one-pot and solvent-free carbonization method, based on the pioneering work of Hsu and co-workers in the use of SCGs as CD precursors [25]. More specifically, the three SCG-based CDs were prepared from different coffee blends from Portuguese brands: Chave D’Ouro^®^ decaffeinated blend, Nicola^®^ Rossio (intense) blend, and Chave D’Ouro^®^ Prestígio (Prestige) blend. The resulting CDs were then identified as: decaf-CDs from Chave D’Ouro^®^ decaffeinated blend, rossio-CDs from Nicola^®^ Rossio (intense) blend and prestige-CDs from Chave D’Ouro^®^ Prestígio (Prestige) blend. 

All CDs were fabricated according to the same procedure. Namely, 2.5 g of each SCG sample were placed in a beaker, which was heated at 200 °C for 4 h in a VWR DL 112 Prime oven (2500 W power consumption). The synthesized CDs were subsequently dissolved in water, while discarding unsuspended and insoluble aggregates. Purification was initially done by centrifuging the aqueous solutions at 6000 rpm for 30 min in order to eliminate suspended impurities. The CDs were further purified by dialysis (Float-A-Lyzer^®^ G2 Dialysis Device, MWCO: 500 Da), which ran continuously for three days. After that, the samples were freeze-dried, and the resulting powder was stored for further use. The CDs synthesized from citric acid and urea (CA@U-CDs) were prepared with the same procedure, by using 2.5 g of citric acid + urea (3:1) as precursor powder instead of SCGs.

### 2.3. Characterization

Zeta potential measurements were determined by using a particle analyzer Anton Paar Litesizer™ 500. X-ray photoelectron spectroscopy (XPS) data were obtained with a Fi Kratos Axis Ultra HAS-VISION. AFM analysis was carried out using a Veeco Metrology Multimode/Nanoscope IVA by tapping. A silica plate was used to deposit the sample for analysis and an AFM RTESP cantilever was used. UV–Vis spectra were obtained with a UV-3100PC spectrophotometer, by using quartz cells. Fluorescence spectra were obtained in standard 10 mm fluorescence quartz cells and collected in a Horiba Jovin Yvon Fluoromax-4 spectrofluorimeter. Spectra were obtained with a 1 nm interval and 5 nm slit widths. The assessment of photostability was made by subjecting CDs to irradiation by a low-pressure lamp (40 W, model Vilber, VL-340.BL) emitting UV radiation at 365 nm.

### 2.4. Calculation of Fluorescence Quantum Yield

The relative fluorescence quantum yield (QY_FL_) was calculated with a standard procedure, [30,31] which is based on the comparison of the integrated luminescence intensities and absorbance values of the relevant sample (different CDs) with that of a reference fluorophore, with the following equation:

(1) $$QY_{FL}^{Sample}=QY_{FL}^{Quinine\;Sulphate}\times \frac{Grad_{Sample}}{Grad_{Quinine\;Sulphate}}\times \frac{\eta_{Sample}^{2}}{\eta_{Quinine\;Sulphate}^{2}}\;$$

In Equation (1), *Grad* is the gradient from the plot of integrated fluorescence intensity versus absorbance and *η* is the refractive index. Quinine sulphate was chosen as a reference fluorophore of known quantum yield (QY_FL_ = 0.54) [32]. Fluorescence was measured with a Horiba Jovin Yvon Fluoromax-4 spectrofluorimeter. Absorbance measurements were made with a UNICAM Helios Gamma, using quartz cells.

### 2.5. Scope and System Boundaries

This was a cradle-to-grave study that aimed to compare the potential environmental impacts of the synthesis of CDs from either waste products (SCGs) from the coffee industry or typical precursors used at laboratory scale. More specifically, the aim of this LCA study was to obtain comparative information regarding the environmental impacts of using SCG samples or typical precursors to fabricate CDs, and not to obtain quantitative data. This work was focused on the manufacturing stage of the target CDs and considered the direct emissions from the production of CDs and indirect impacts associated with upstream resource extraction and energy generation.

This work used thermal carbonization of three different SCGs and one citric acid—urea mixture—as the synthetic strategy for the four studied CDs. A detailed description of the four routes can be found in Section 2.2. The environmental impacts were analyzed and compared in the first stage using a weight-based functional unit of 1 kg of CDs. This was made because the selection of a weight-based functional unit makes sense when comparing the production of an equivalent nanomaterial [33,34]. However, these impacts were normalized by the QY_FL_ of the different CDs (Table 2), in a later stage. Such an approach was required because weight-based functional units do not consider the functional benefits of the materials for which they were engineered, and so, they do not account for the possibility that a more resource-intensive synthesis may be later justified in the use stage (given the improved functionality). QY_FL_ was chosen as a normalization factor because, while CDs have many different applications, a high QY_FL_ is a required property in most, if not all, of these applications. In fact, a high QY_FL_ is essential for both imaging and sensing applications, which are the practical applications developed to date for SCG-based CDs.

### 2.6. Life Cycle Inventory Data

The assessment of the environmental impacts associated with the fabrication of CDs (via thermal carbonization) was based on inventory (foreground) data from laboratory-scale synthesis procedures performed by our group, as described in Section 2.2. The life cycle inventory data for the foreground system consisted of average data found on the Ecoinvent^®^ 3.5 database for the synthesis procedures (primary data).

The present LCA model only included the carbonization step, given that this was a comparative study, and not a quantitative one. Subsequent purification steps were the same for all CDs, and so, they should have provided the same impacts for all synthesis procedures. Thus, our model only included the electricity used to power the oven during the carbonization step (10 kWh) and the number of precursors needed to produce 1 kg of CDs, which was calculated considering the synthesis yields described (Table 1). More specifically, CA@U-CDs required 7.61 kg of citric acid and 2.54 kg of urea (synthesis yield of 9.85% and a 3:1 ratio) to produce 1 kg of CA@U-CDs. As for the SCG samples, it should be noted that in LCA terms, their use as CD precursors should not generate environmental impacts associated with their production. Namely, SCG samples are produced irrespective of their subsequent use as CDs precursors, given their production results entirely from coffee brewing. Thus, any environmental impact resulting from the production of SCG samples should be attributed to the coffee industry, and not to the production of CDs. Nevertheless, all four CDs produced unspecified chemical waste that should be accounted for in the final waste flows: 9.2 kg for CA@U-CDs, 187.7 kg for decaf-CDs, 130.6 kg for rossio-CDs, and 50.0 kg for prestige-CDs. Thus, our LCA model regarding the synthesis of SCG-based CDs only contemplated the electricity used to power the oven and the final waste flows, while for CA@U-CDs the electricity, final waste flows and the amounts of citric acid/urea were considered. 

The different processes and chemicals included in this study were modeled with the following life cycle inventory (LCI) data: citric acid {GLO} | market for; Urea, as N {GLO} | market for; Electricity, medium voltage {PT} | market for; Chemical waste, unspecified. The amounts of citric acid and urea are described above in the “Fabrication of CDs” section. The electricity process refers to the electricity used by the oven (VWR DL 112 Prime) during the carbonization process (200 °C for 4 h). This oven possesses a capacity of 112 L and a nominal power consumption of 2500 W. This results in an electricity amount of 10 kWh for each synthesis.

### 2.7. Environmental Impact Assessment 

LCA studies were performed using SimaPro 9.0.0.35 Multiuser software. Environmental impacts were modeled using the ReCiPe 2016 v1.1 LCIA, Hierarchist version [35]. The impact potentials evaluated according to the ReCiPe method were: global warming—human health (GW–HH), global warming—terrestrial ecosystems (GW–TE), global warming—freshwater ecosystems (GW–FE), stratospheric ozone depletion (SO), ionization radiation (IR), ozone formation—human health (OF–HH), fine particulate matter formation (FPM), ozone formation—terrestrial ecosystems (OF–TE), terrestrial acidification (TA), freshwater eutrophication (FE), marine eutrophication (ME), terrestrial ecotoxicity (TE), freshwater ecotoxicity (TET), marine ecotoxicity (MET), human carcinogenic toxicity (HC), human non-carcinogenic toxicity (HNC), land use (LU), mineral resource scarcity (MR), fossil resource scarcity (FR), water consumption—human health (WC–HH), water consumption—terrestrial ecosystem (WC–TE) and water consumption—aquatic ecosystems (WC–AE).

### 2.8. Sensitivity Analysis

A sensitivity analysis was performed to help to better understand uncertainties associated with this study by considering “what if” scenarios [36]. That is, by varying the amount and type of materials and electricity employed in the synthesis of the CDs. In this way, it is possible to evaluate to what extent key assumptions affect LCA results.

## 3. Results and Discussion

### 3.1. Synthesis of CDs

**Figure 1:**
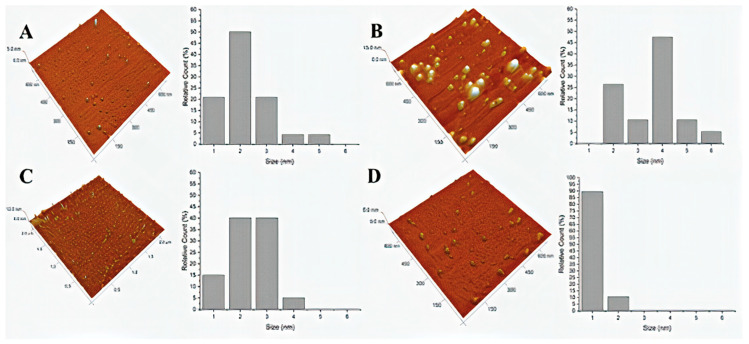
Atomic force microscopy (AFM) images and relative size distribution for (**A**) decaf-CDs, (**B**), rossio-CDs, (**C**) prestige-CDs and (**D**) CA@U-CDs.

The thermal carbonization of the three SCG samples led to the formation of nanoparticles with a relatively heterogenous size distribution (Figure 1). These measurements also allowed us to obtain the following average sizes for the SCG-based CDs (± standard deviation): 2.1 ± 1.0 nm for decaf-CDs, 3.9 ± 1.0 nm for rossio-CDs and 2.3 ± 0.8 nm for prestige-CDs. These values are in line with the sizes between 1 and 5 nm obtained in previous studies focused on the preparation of CDs from SCGs [25,26,27,28]. The thermal carbonization of citric acid + urea lead to the formation of CDs with a homogeneous distribution, with an average size of 1.0 ± 0.3 nm (Figure 1).

Reaction yields (mass by mass) were obtained by measuring the weight of the freeze-dried powder relative to each sample, after steps of solubilization, centrifugation and dialysis. Unfortunately, the reaction yields for the reactions using the three SCG samples as precursors were not satisfactory: 0.53% for decaf-CDs, 0.76% for rossio-CDs and 1.96% for prestige-CDs (Table 1). Nevertheless, these values are in line with those obtained (2%) for the production of CDs from Nescafe^®^ original instant coffee [26]. Furthermore, while the reaction yield for CA@U-CDs was higher (9.85%), this is still a rather poor yield (Table 1). Thus, it appears that in terms of reaction yield, the efficiency of the synthesis using SCGs is comparable to that of the same process using standard precursors. It should also be noted that the yields of the production of CDs are generally quite low [37,38,39]. Given this, the problem of low production yield cannot be ascribed specifically to SCG-based CDs, but to CDs in general. Thus, the main conclusion that can be obtained from these results is that SCG-based CDs present similar production yields to typical CDs.

In conclusion, these results demonstrate that the thermal treatment of SCG samples led to the formation of nanoparticles with a yield similar to that of using standard precursors. Further evaluation and characterization were then done to assess if the resulting CDs possessed potentially advantageous characteristics.

### 3.2. Surface Characterization of CDs

An XPS analysis was done to analyze the surface composition of the four CD samples (Figures S1–S3). More specifically, the XPS atomic composition (at w %) of each sample was determined (Table 1). It was revealed that all types of sample (both SCG-derived and CA@U-CDs) were composed mostly of C (~57–58%) and O (~27–35%). The main differences were the presence of small amounts of K (~2–3%) in the SCG-derived CDs, and that the N molecule fraction in CA@U-CDs (~14%) was significantly higher than in the SCG-based CDs (~3–6%). This lower N (at w %) in the latter samples was related with higher amounts of O. A detailed scan for the internal levels of C 1s, O 1s and N 1s was subsequently done, towards deconvolution and chemical state and the quantitative analysis (Figures S1–S3).

C 1s spectra could be split into four peaks for all samples: at binding energies of ~284 eV (attributed to C-C/C-H groups) [40], ~286 eV (C-O/C-N groups) [41], ~287 eV (C=O groups) and ~288 eV (O-C=O) [42]. SCG-derived decaf-CD and prestige-CD samples presented a very similar composition, with a higher contribution from C-C/C-H groups (~47–48%), followed by C-O/C-N groups (~36-38%). The contribution by C=O groups was moderate (~11%), while that by O-C=O groups was negligible (~3%). This analysis might indicate a small degree of π-conjugation at the surface of these CDs, with potential impacts on the photoluminescence efficiency. The degree of π-conjugation should be slightly higher for SCG-derived rossio-CDs, given a reduced contribution of C-C/C-H groups (~40%), and the consequent higher contribution of C-O/C-N (~42%) and C=O (~16%) groups. Nevertheless, the contribution by C 1s was qualitatively very similar between all SCG-based CDs. This is not true, however, for CA@U-CDs. For these CDs, the contribution of π-conjugated groups (C=O and O-C=O) was 35%, with an emphasis on O-C=O groups (~25%), while that contribution was just ~15% for SCG-based CDs. This increase resulted from a decrease in the contribution of C-N/C-O groups (~17%), while the contribution by C-C/C-H groups remained predominant (~47%). C 1s analysis indicated that the degree of π-conjugation at the surface of CA@U-CDs should be significantly higher than at the surface of SCG-based CDs.

O 1s could be split into just two peaks: at binding energies of ~531 eV (C=O groups) and ~532 eV (C-O groups) [42]. The three SCG-based CDs presented identical compositions: predominant contributions by C-O groups (~67–74%), with moderate contributions from C=O groups (~25–32%). The composition of CA@U-CDs was quite different, however, given that the dominant contribution was from C=O groups (~84%), while the contribution by C-O groups was quite moderate (~16%). These results further support the notion that the degree of π-conjugation at the surface of CA@U-CDs was higher than at the surface of CA@U-CDs, with potential benefits for the former regarding their photoluminescent properties. Finally, N 1s could also be split into two peaks: at binding energies of ~400 eV (amine/amide groups) and ~401.4 eV (protonated amides). All four samples presented dominant contributions from amine/amide groups (~88–95%), with small/almost negligible contributions from protonated amines (~4–12%) [43]. 

The zeta potential of all samples was measured by DLS in aqueous solutions, revealing the following results: −24 mV for decaf-CDs, −12.5 mV for rossio-CDs, −18.1 mV for prestige-CDs and −0.5 mV for CA@U-CDs. This indicates the possibility that the SCG-based CDs were negatively charged, while CA@U-CDs should have presented a neutral state.

### 3.3. Fluorescent Characterization of CDs

**Figure 2:**
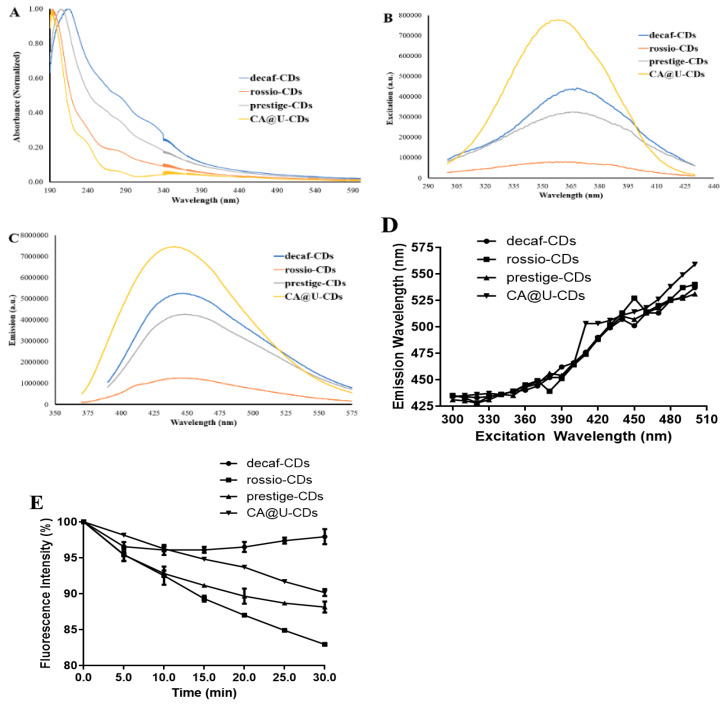
(**A**) Absorption, (**B**) excitation and (**C**) fluorescence spectra in aqueous solution for decaf-CDs, rossio-CDs, prestige-CDs and CA@U-CDs. (**D**) Emission wavelength (in nm) as a function of the excitation wavelength (in nm). (**E**) Photostability of the four CDs, measured as the variation of the fluorescence intensity as a function of irradiation time under a UV light source (365 nm).

The absorption, excitation and fluorescence spectra of the four samples can be found in Figure 2. The two latter spectra were obtained at the fluorescent and excitation wavelength maxima, respectively.

All samples presented the typical absorption spectra of CDs (Figure 2A), with strong absorbance in the UV region and the absence of the comparable absorbance of the wavelengths of the excitation maxima [44,45]. All four CDs present quite similar excitation wavelength maxima (Figure 2B): 370 nm for decaf-CDs and prestige-CDs, 360 nm for rossio-CDs and 350 nm for CA@U-CDs. The same can also be said for their fluorescence maxima, given that all CDs generated blue emission (Figure 2C): 450 nm for decaf-CDs, 445 nm for rossio-CDs, 450 nm for prestige-CDs and 440 nm for CA@U-CDs. This blue emission was consistent with the fluorescence exhibited by other SCG-based CDs described in the literature [25,26,27]. This indicates that SCG samples can be used to produce CDs with comparable emission and excitation maxima to those obtained by using typical precursors. However, it is noticeable that the bands of CA@U-CDs were more well-resolved than those of SCG-derived CDs, especially considering their excitation spectra. The broader excitation bands of SCG-derived CDs could be attributed to their higher size heterogeneity. 

Interestingly, all CD samples presented an excitation-dependent emission (Figure 2D), which is a typical feature of CDs [46,47]. Such a feature has also been found in the case of SCG-based CDs [25,26,27]. Namely, the emission maxima of these four nanoparticles is in the blue region of the visible spectrum. However, increasing the excitation wavelength from 300 to 500 nm, leads to a quite significant ~100 nm redshift in the emission. Therefore, SCG-derived CDs can also emit green light, albeit with less efficiency than blue fluorescence.

Given the relative size heterogeneity of the SCG-based CDs (Figure 1), one could be tempted to attribute this excitation-dependence to quantum confinement effects [48], that is, particles of different sizes emit with different energies. However, the size homogeneity of CA@U-CDs do not support this hypothesis. Thus, it is possible that this excitation-dependence arises from sp^2^ subregions in the carbon core and from functional groups on the surface, as hypothesized by Van Dam et al. [47]. Nevertheless, the luminescence of CDs should not be explained by just a single dominating mechanism, but rather by two or more processes (such as quantum size effects, bandgap transitions or defect-derived emission) [49].

**Figure 3:**
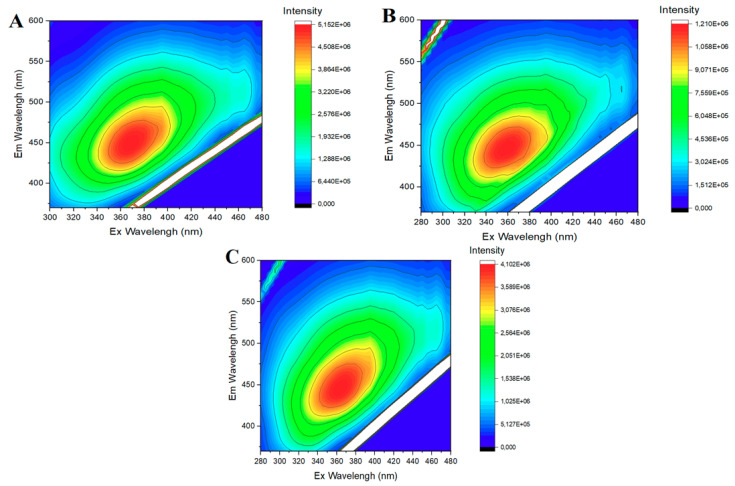
Two-dimensional excitation-emission matrices (EEMs) for decaf-CDs (**A**), rossio-CDs (**B**) and prestige-CDs (**C**).

Such a conclusion is supported by the excitation–emission (EEM) matrices of the three SCG-based CDs, which are presented as 2D color surfaces in Figure 3. These EEMs show only a well-defined luminescent region for each SCG-based CD, with a maximum in the blue region of the spectrum, and similar luminescent centers (which could be expected if the excitation-dependent emission was the result of different emitter species mixed in the solution).

The QY_FL_ for the four CD samples can be found in Table 1. The QY_FL_ obtained for SCG-based CDs is interesting (between 2.9 and 5.8%), thereby indicating that SCGs can be appropriate precursors for the fabrication of CDs. Such values are in line with those obtained for other SCG-based CDs [25,26,27], which were between 3.8% and 8%. However, SCG-based CDs appear to be at disadvantage when we compare them directly with CDs prepared with typical precursors. Namely, the QY_FL_ of CA@U-CDs was significantly higher (22.5%) than those recorded by CDs derived from SCGs. Two reasons can explain the higher QY_FL_ of CA@U-CDs: one, the higher π-conjugation of the surface of this type of CD as demonstrated by XPS analysis (Figures S1–S3); the second reason is the higher N XPS mole fraction of this type of CD, also demonstrated by XPS analysis. More specifically, the N-doping of CDs has been demonstrated to generate a high QY_FL_ [50,51]. Additionally, the lower content of amide groups on the surface of CDs vs. CA@U-CDs (see XPS N content) may also account for the observed differences. In the synthesized model CDs, the abundance of amide groups on the surface favors photoinduced charge transfer between amide and carboxylic acid groups via hydrogen bond interactions, enabling a more rigid structure, with this being the origin of the high blue fluorescence observed in almost all CDs [52]. 

Figure 2E shows the photostability of the four CD samples, which was accounted for by measuring their fluorescence after subjecting them to UV radiation at 365 nm (low pressure UV lamp, 40 W). Decaf-CDs were the most photostable CDs, with their fluorescent intensity only decreasing by ~3% in a 30-min period. Prestige-CDs and CA@U-CDs presented similar photostability, as their fluorescence intensity decreased by ~10% in the same 30-min period. Rossio-CDs were shown to be the least photostable, with a decrease of ~20% in the measured time period. Given this, this analysis revealed that at least decaf-CDs and prestige-CDs CDs can be considered to be photostable (with decreases of just ~3% and ~10% when subjected to 30 min of UV irradiation). Moreover, these SCG-based CDs presented comparable photostability to that of typical CDs, such as CA@U-CDs.

### 3.4. Sensing of Fe^3+^

Arguably, one of the most common and well-studied applications for CDs is their use as fluorescent probes in the sensing of heavy metal cations [53,54,55]. The focus on this field results from the significant adverse health effects resulting from the presence of heavy metals in water resources. One of such metal cations is Fe^3+^ [53], as iron is an essential trace element for human metabolism, but elevated intake of this metal can lead to health hazards due to the production of reactive oxygen species (ROS) [53]. ROS can cause several diseases, such as Alzheimer’s and Parkinson’s diseases and cancer [56]. United States Environmental Protection Agency (USEPA) established a permissible limit of 0.3 mg/L (5.357 μM) in drinking water [53].

**Figure 4:**
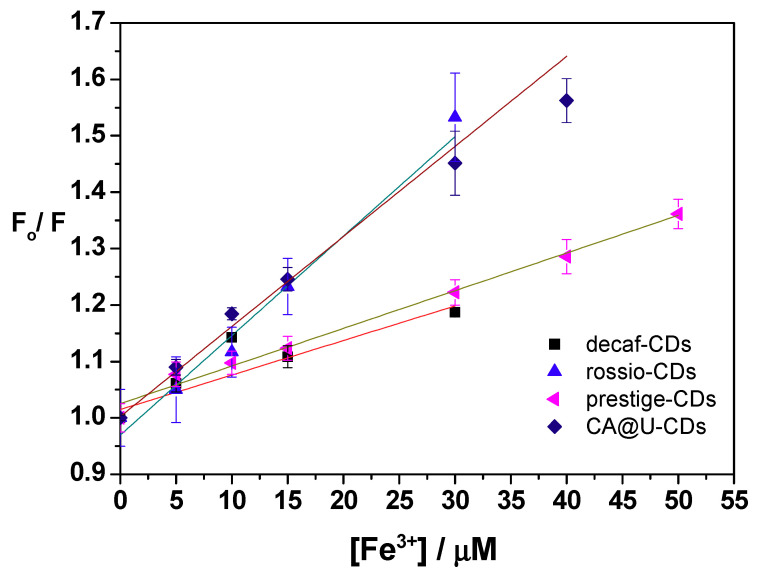
Emission profiles of (■) decaf-CDs, (◄) prestige-CDs, (♦) CA@U-CDs (▲) and rossio-CDs (d) as a function of the concentration of Fe^3+^ (in mM), in aqueous solution. F_0_ and F are the relative fluorescence intensities in the absence and presence of Fe^3+^, respectively. R^2^ = 0.85; 0.98; 0.98 and 0.96, respectively.

The potential Fe^3+^-induced quenching of SCG-based CDs was subsequently explored, with the emission profiles of CDs as a function of Fe^3+^ presented in Figure 4. Fe^3+^ induced a concentration-dependent quenching for all four CD samples, with quenching following a linear Stern–Volmer relationship. However, an R^2^ of 0.82 for decaf-CDs indicates the inability of this type of SCG-based CDs to be used as a fluorescent probe for Fe^3+^. In contrast, an R^2^ of 0.99 for the other CDs indicate their potential as fluorescent probes for this heavy metal. Fitting the data of decaf-CDs, rossio-CDs, prestige-CDs and CA@U-CDs to the standard Stern–Volmer equation revealed the following Stern–Volmer constants (K_SV_): 5.80 × 10^−3^, 1.82 × 10^−2^, 6.71 × 10^−3^ and 1.39 × 10^−2^ μM^−1^, respectively. These values correlate well with the obtained quenching for each CD: 15.8% for decaf-CDs, 34.8% for rossio-CDs, 26.5% for prestige-CDs and 36.0% for CA@U-CDs. The emission profiles (Figure 4) also allowed us to obtain limits of detection (LoD) for each CD: 14.11 μM for decaf-CDs, 3.51 μM for rossio-CDs, 4.58 μM for prestige-CDs, and 3.07 μM for CA@U-CDs.

These results pointed to SCG-based CDs (except for decaf-CDs) exhibiting a response to Fe^3+^ comparable to CA@U-CDs. Furthermore, their LoDs were below the permissible limit for Fe^3+^ (5.357 μM) in drinking water, as established by USEPA [52]. Both rossio-CDs and prestige-CDs have the potential to be employed as fluorescent probes of environmental relevance, with a response to Fe^3+^ comparable to that shown by CDs prepared with typical precursors (CA@U-CDs).

### 3.5. Comparative LCA Study

Another parameter that should be considered while evaluating the potential of certain samples as CD precursors is the environmental impact they cause by using them in the synthesis and development of CDs. That is, new precursors should lead to a sustainable synthesis strategy for this type of nanoparticle. This is particularly important since the production steps of engineered nanomaterials have been identified as being of environmental concern [57]. In fact, it has been found that the production of engineered nanomaterials can be orders of magnitude more energy- and material-consuming than for fine chemicals and pharmaceuticals [58]. Furthermore, LCA studies of engineered nanomaterials have shown that the energy and chemicals used during the synthesis of these nanomaterials can contribute a significant share of the environmental impacts generated during their life cycle.

To obtain comparative information regarding the sustainability of using SCG samples as CD precursors we have performed an LCA study, given that LCA aims to quantify the relevant environmental impacts of a given system during its life cycle [13,59,60,61,62]. To this end, the environmental impacts of synthesizing CDs with selected precursors (decaf-CDs, rossio-CDs and prestige-CDs) and typical reagents (CA@U-CDs) was analyzed and compared by using a mass-based functional unit of 1 kg of CDs. This was made because the selection of a weight-based functional unit (FU) makes sense to compare the production of an equivalent amount of nanomaterial [33]. However, these impacts were later normalized by the QY_FL_ of the CDs. Such an approach is needed because weight-based functional units do not consider the functional benefits of the engineered nanomaterials for which they were fabricated, and so might not account for the possibility that a more resource-intensive synthesis may be justified later in the use stage (given the improved functionality).

**Figure 5:**
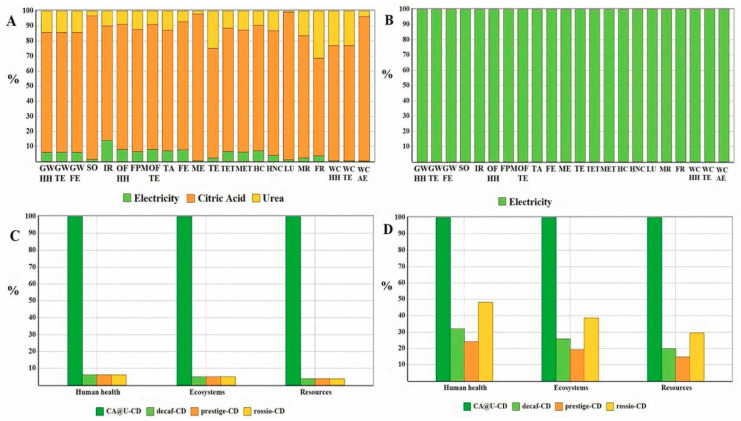
Relative environmental impacts of CA@U-CDs (**A**) and of spent coffee grounds (SCG)-based CDs (**B**) with the ReCiPe2016 as the life cycle impact assessment (LCIA) method. Comparative environmental (**C**,**D**) profiles for the four different routes for the synthesis of CDs. Environmental profiles were obtained using ReCiPe2016 as LCIA. Graphic C refers to an analysis using a weight-based functional unit of 1 kg of CDs, while Graphic D is re-scaled with respect to the QY_FL_ of the CDs.

The relative environmental impacts associated with the synthesis of the four CDs are presented in Figure 5, by considering a weight-based functional. The analysis of the synthesis of CA@U-CDs (Figure 5A) shows that citric acid is the main agent responsible for the resulting environmental impacts in all categories, followed by urea. The contribution made by the use of electricity is always below 10%, except in the case of ionization radiation. The use of electricity is the only contributing parameter for the synthesis of SCG-based CDs (Figure 5B), given that it is the only input parameter in their synthesis. This results from the fact that, as already referred to in Section 2.6, the use of SCGs as CD precursors should not generate environmental impacts associated with their production, given that their production results entirely from coffee brewing. More specifically, SCG samples are produced irrespective of their subsequent use as CD precursors, and so any environmental impact resulting from the production of SCG samples should be attributed to the coffee industry.

All four CDs are compared in Figure 5C, with all weight-based environmental categories being divided into three main ones: human health, ecosystems and resources. It is clearly seen that CA@U-CDs produce significantly more environmental impacts in all categories than any of the SCG-derived CDs. This results from the significant use of citric acid and urea as precursors, while the latter CDs use a waste product of another industry as a precursor. Furthermore, this LCA assessment does not detect any difference regarding the environmental impacts produced by any SCG-based CD. This is not surprising, given that all CDs use the same amount of electricity.

The final step of this study was to assess the environmental impacts associated with each synthetic route, when rescaled with respect to the QY_FL_ of the resulting CDs (Table 2 and Figure 5D). This rescaling was done by considering the highest QY_FL_ (that of CA@U-CDs) as the reference QY_FL_ ($QY_{FL}^{REF}$). The QY_FL_-normalized functional unit for each CD was then calculated as $\frac{QY_{FL}^{REF}}{QY_{FL}}$ (Table 2).

There were not many qualitative differences with the rescaling, given that the fabrication of CA@U-CDs still produced significantly higher environmental impacts in all categories than any of the SCG-based CDs, even when it was considered a function-based functional unit. However, the contributions from each SCG-based CD was significantly higher in all three categories: decaf-CDs (19.7–32.1%), rossio-CDs (29.6–48.1%) and prestige-CDs (14.8–24.1%). From the three categories, the one associated with higher impacts is that of human health, followed by ecosystems and then by resources. Finally, for a QY_FL_-based functional unit, the SCG-based CDs that generated higher environmental impacts were rossio-CDs > decaf-CDs > prestige-CDs. 

A sensitivity analysis was performed by changing the input of the electricity and raw materials (citric acid and urea) by ± 30%. The results for changing the input of citric acid to 70% and 130% of the original value are present in Figure S4. This variation did not lead to any significant qualitative change, given that it still was CA@U-CDs that produced the highest environmental impacts in all categories (by a wide margin). Nevertheless, these results indicate that changing the amount of citric acid does have a non-negligible quantitative impact, as reducing the amount of citric acid by 30% led to a ~10% approximation between the impacts produced by CA@U-CDs and SCG-based CDs. In turn, a 30% increase led to a ~10% distancing.

The comparative environmental profiles for all CDs when changing the input of urea to 70% and 130% of its original value is shown in Figure S5. Changing the amount of urea did not lead to either significant qualitative or quantitative effects. CA@U-CDs still led to the higher environmental impacts, and the contribution of SCG-based CDs was nearly unchanged in all three categories.

Finally, the comparative environmental profiles for different inputs of electricity can be found in Figure S6. Once again, changing the amount of electricity used did not change the fact that CA@U-CDs led to higher environmental impacts, when in comparison with the studied SCG-based CDs. Nevertheless, varying the amount of electricity did have a non-negligible effect in the three categories. This effect is similar to that exerted by varying citric acid, but in an opposite way. That is, decreasing the input of electricity by 30% led to a ~10% distancing between CA@U-CDs and SCG-based CDs (while the same variation of citric acid led to an approximation), while increasing the electricity by 30% led to a ~10% approximation (while the reverse was true for citric acid). Thus, decreasing the used electricity favored the sustainability of SCG-based CDs, while decreasing the amount of citric acid favored CA@U-CDs. Urea did not seem to provide a significant effect either way.

In summary, SCG samples, despite lower synthesis yields and QY_FL_, are a better option than typical precursors (citric acid and urea) for the fabrication of CDs in terms of environmental impacts. This is true both when considering a weight-based and a function-related functional unit. Prestige-CDs should be the preferred option when considering their QY_FL_.

## 4. Conclusions

The fabrication of fluorescent CDs by using either spent coffee grounds (SCGs, biomass waste material from the coffee industry) or standard precursors (citric acid and urea) as precursors was compared, both in terms of associated environmental impacts and the structural and luminescent properties of these nanoparticles. The one-pot and solvent-free carbonization of the different precursor samples led to the formation of nanoparticles with an average size of 1.0–3.9 nm and a similar blue emission. However, while SCG-based CDs present moderate quantum yields (2.9–5.8%) and quite low reaction yields, CDs made from citric acid and urea showed more a significant quantum yield (22.5%) and higher reaction yields. Nevertheless, an LCA study demonstrated that the fabrication of CDs with SCGs is more environmentally beneficial than using typical precursors, irrespective of using either a weight-based or function-related functional unit. Thus, while comparison with CDs generated with typical precursors indicates that SCG-based CDs could benefit from some improvement, the present study validates the use of SCGs (instead of more common organic molecules) as precursors as the most sustainable route for the fabrication of CDs. SCG-based CDs also demonstrated their potential as sensing probes for Fe^3+^ in water.

## Figures and Tables

**Table 1:** Atomic surface compositions (%) *, synthesis yields (%), fluorescence quantum yield (QY_FL_) (%) for decaf-carbon dots (CDs), rossio-CDs, prestige-CDs and CA@U-CDs.

	Decaf-CDs	Rossio-CDs	Prestige-CDs	CA@U-CDs
C (%)	59.1	57.1	59.0	58.9
O (%)	35.3	34.9	34.1	27.2
N (%)	2.8	6.2	4.4	13.9
K (%)	2.7	1.8	2.5	-
Synthesis yield (%)	0.53	0.76	1.96	9.85
QY_FL_ (%)	4.3	2.9	5.8	22.5

* Obtained by X-ray photoelectron spectroscopy (XPS).

**Table 2:** Function-related functional unit for decaf-CDs, rossio-CDs, prestige-CDs and CA@U-CDs.

Function-Related Functional Unit	Decaf-CDs	Rossio-CDs	Prestige-CDs	CA@U-CDs
$\frac{QY_{FL}^{REF}}{QY_{FL}}$	5.2	7.8	3.9	1.0

## Supplementary Materials

The following are available online at https://www.mdpi.com/2079-4991/10/6/1209/s1, Figure S1: XPS C 1s spectra for decaf-CDs (**A**), rossio-CDs (**B**), prestige-CDs (**C**) and CA@U-CDs (**D**), Figure S2: XPS O 1s for decaf-CDs (**A**), rossio-CDs (**B**), prestige-CDs (**C**) and CA@U-CDs (**D**), Figure S3: XPS N 1s spectra for decaf-CDs (**A**), rossio-CDs (**B**), prestige-CDs (**C**) and CA@U-CDs (**D**), Figure S4: Comparative environmental profiles for the synthesis of all CDs, rescaled with respect to the QY_FL_ of the CDs, by changing the input of citric acid to 70% (**A**) and to 130% (**B**) of the original value, Figure S5: Comparative environmental profiles for the synthesis of all CDs, rescaled with respect to the QY_FL_ of the CDs, by changing the input of urea to 70% (**A**) and to 130% (**B**) of the original value, Figure S6: Comparative environmental profiles for the synthesis of all CDs, rescaled with respect to the QY_FL_ of the CDs, by changing the input of electricity to 70% (**A**) and to 130% (**B**) of the original value.
