# Supplementary material for: Turning Spent Coffee Grounds into Sustainable Precursors for the Fabrication of Carbon Dots
Source: Nanomaterials (Basel). 2020 Jun 21;10(6):1209. doi: 10.3390/nano10061209 (PMC7353129; doi:10.3390/nano10061209)
Supplement: Supplementary file 1 [file nanomaterials-10-01209-s001.pdf]

SUPPORTING INFORMATION

Turning Spent Coffee Grounds into Sustainable Precursors for the Fabrication of Carbon Dots

Diana M.A. Crista [1], Abderrahim El Mragui [2], Manuel Algarra [2,*], Joaquim C.G. Esteves da Silva [1,3], Rafael Luque [4,5,*] and Luís Pinto da Silva [1,3,*]

1 Chemistry Research Unit (CIQUP), Faculty of Sciences of University of Porto, R. Campo Alegre 697, 4169-007 Porto, Portugal; dianacrista4@gmail.com (D.M.A.C.); jcsilva@fc.up.pt (J.C.G.E.d.S.)

2 Department of Inorganic Chemistry. Faculty of Science. University of Málaga. Campus de Teatino s/n 29071. Málaga, Spain; a.elmragui@edu.umi.ac.ma

3 LACOMEPHI, GreenUPorto, Department of Geosciences, Environment and Territorial Planning, Faculty of Sciences of University of Porto, R. Campo Alegre 697, 4169-007 Porto, Portugal

4 Departamento de Química Orgánica, Universidad de Córdoba, Edif. Marie Curie, Ctra N-IVA Km 396, E-14014 Córdoba, Spain

5 Center for Molecular Design and Synthesis of Innovative compounds for Medicine, Peoples Friendship University of Russia (RUDN University), 6 Miklukho Maklaya Street, 117198 Moscow, Russia

* Correspondence: manuel.gonzalez@staff.uma.pt (M.A.); q62alsor@uco.es (R.L.); luis.silva@fc.up.pt (L.P.d.S.)

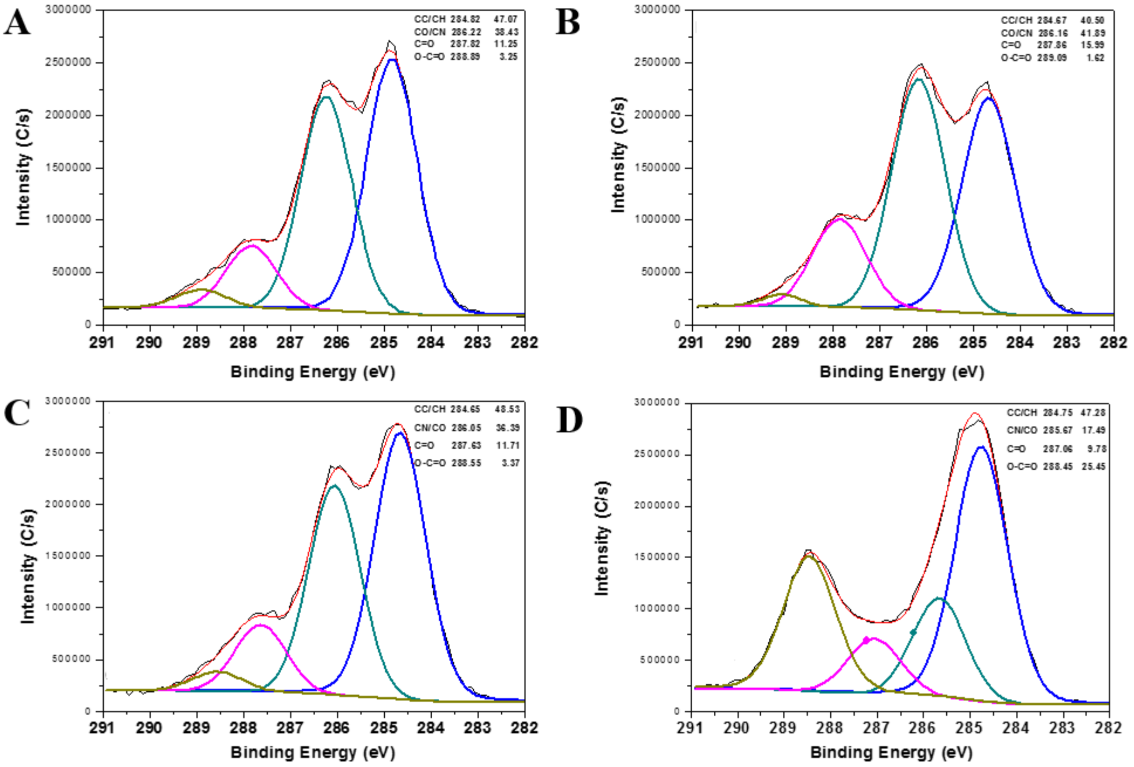

Figure S1 – XPS C 1*s* spectra for decaf-CDs (A), rossio-CDs (B), prestige-CDs (c) and CA@U-CDs (D).

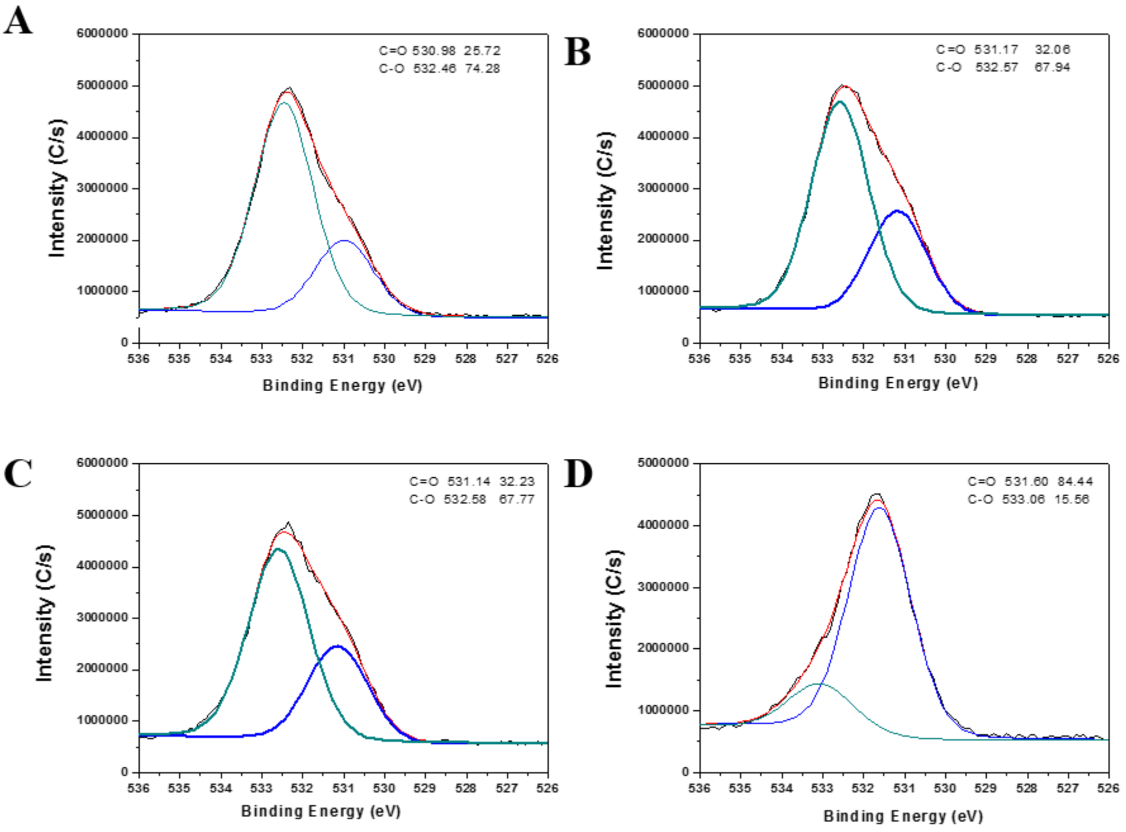

Figure S2 – XPS O 1*s* spectra for decaf-CDs (A), rossio-CDs (B), prestige-CDs (c) and CA@U-CDs (D).

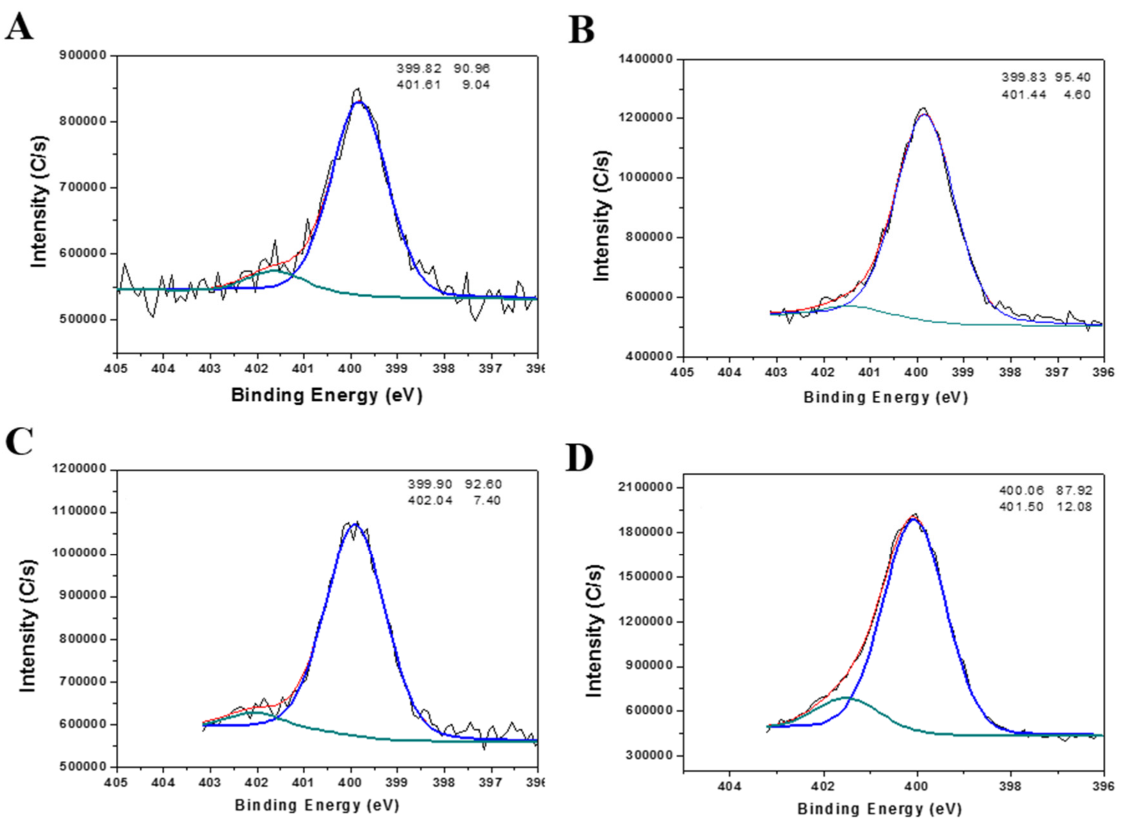

Figure S3 – XPS N 1*s* spectra for decaf-CDs (A), rossio-CDs (B), prestige-CDs (c) and CA@U-CDs (D).

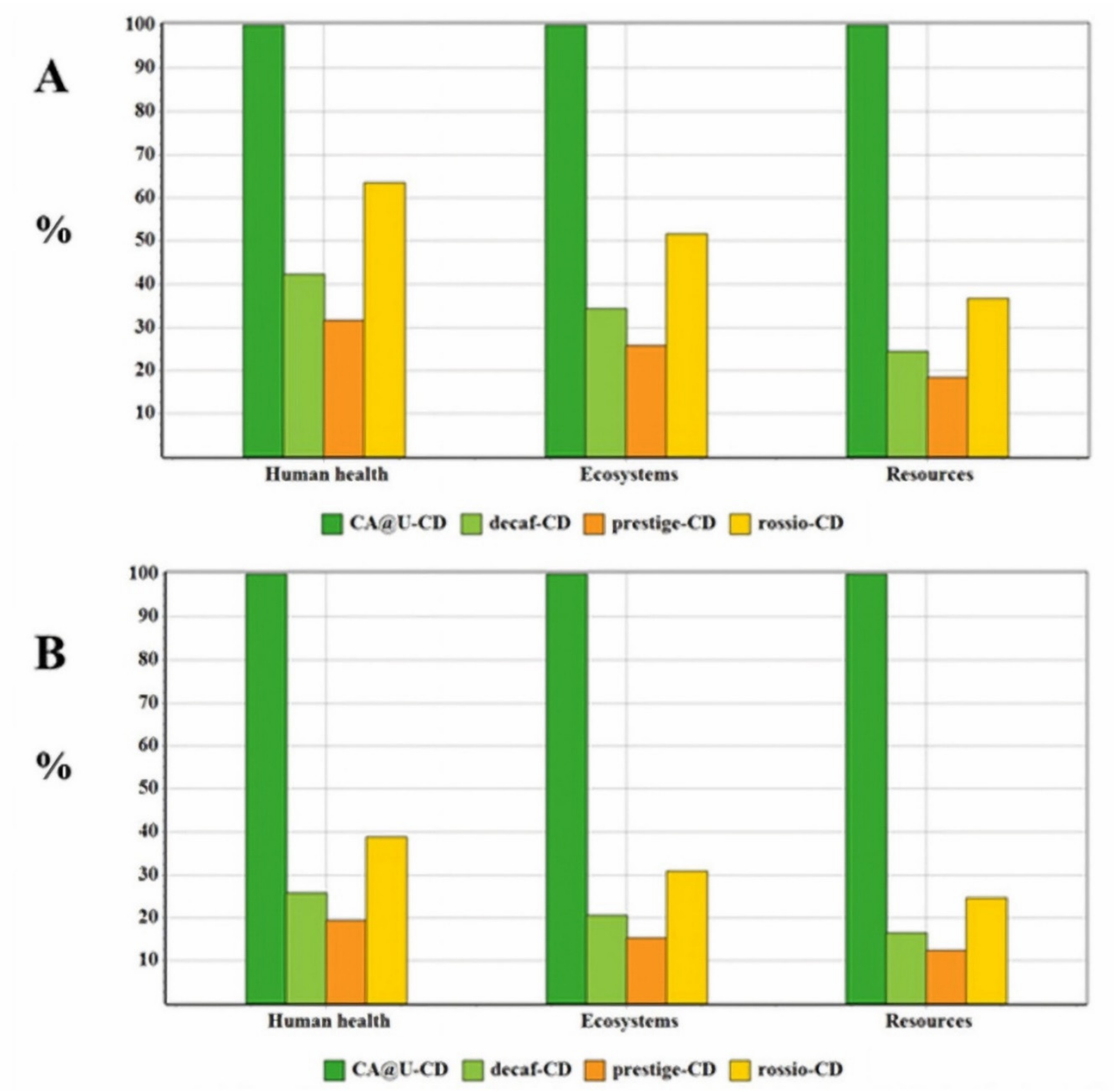

Figure S4. Comparative environmental profiles for the synthesis of all CDs, re-scaled with respect to the QY_{FL} of the CDs, by changing the input of citric acid to 70% (A) and to 130% (B) of the original value.

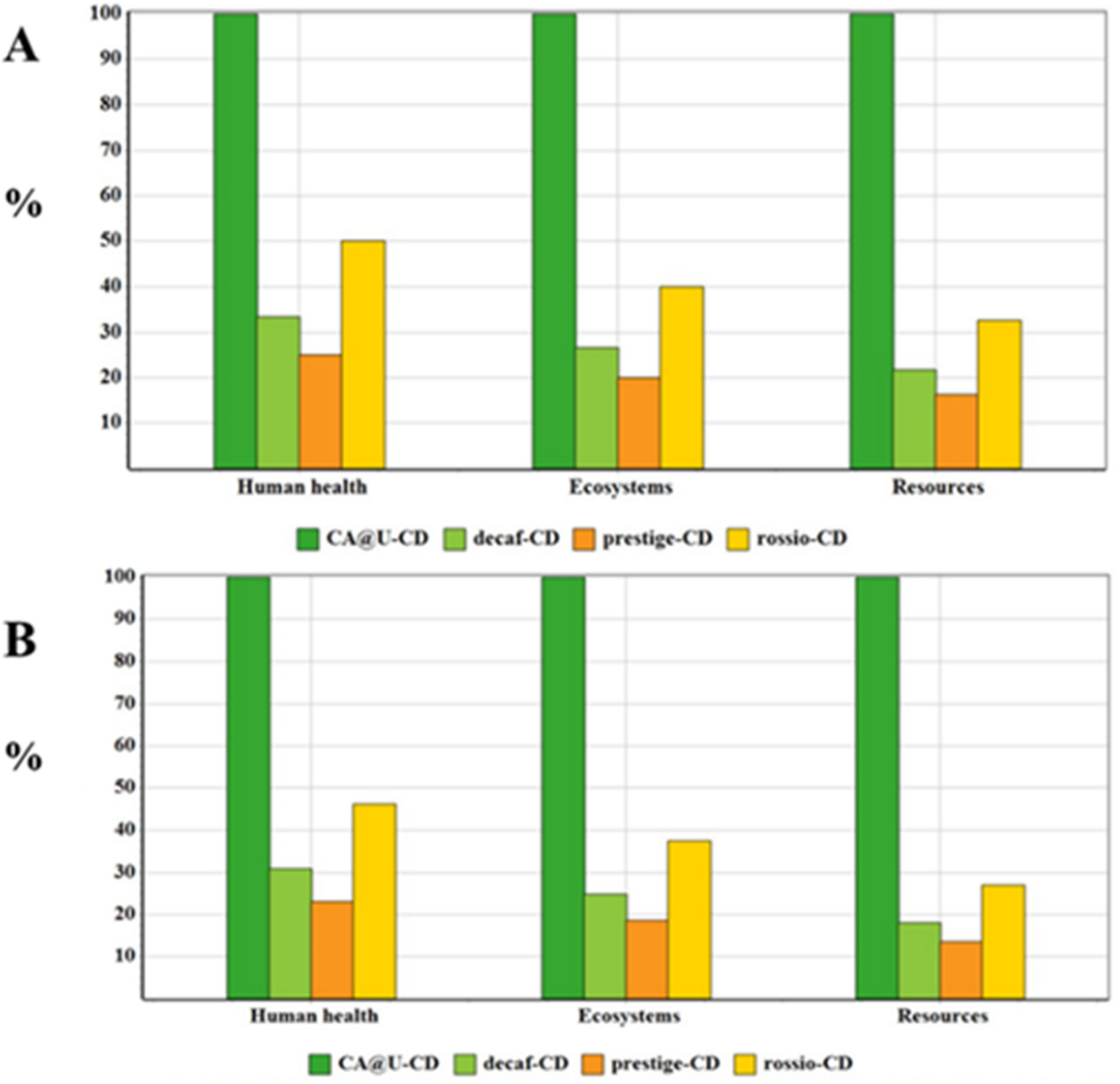

Figure S5. Comparative environmental profiles for the synthesis of all CDs, re-scaled with respect to the QY_{FL} of the CDs, by changing the input of urea to 70% (A) and to 130% (B) of the original value.

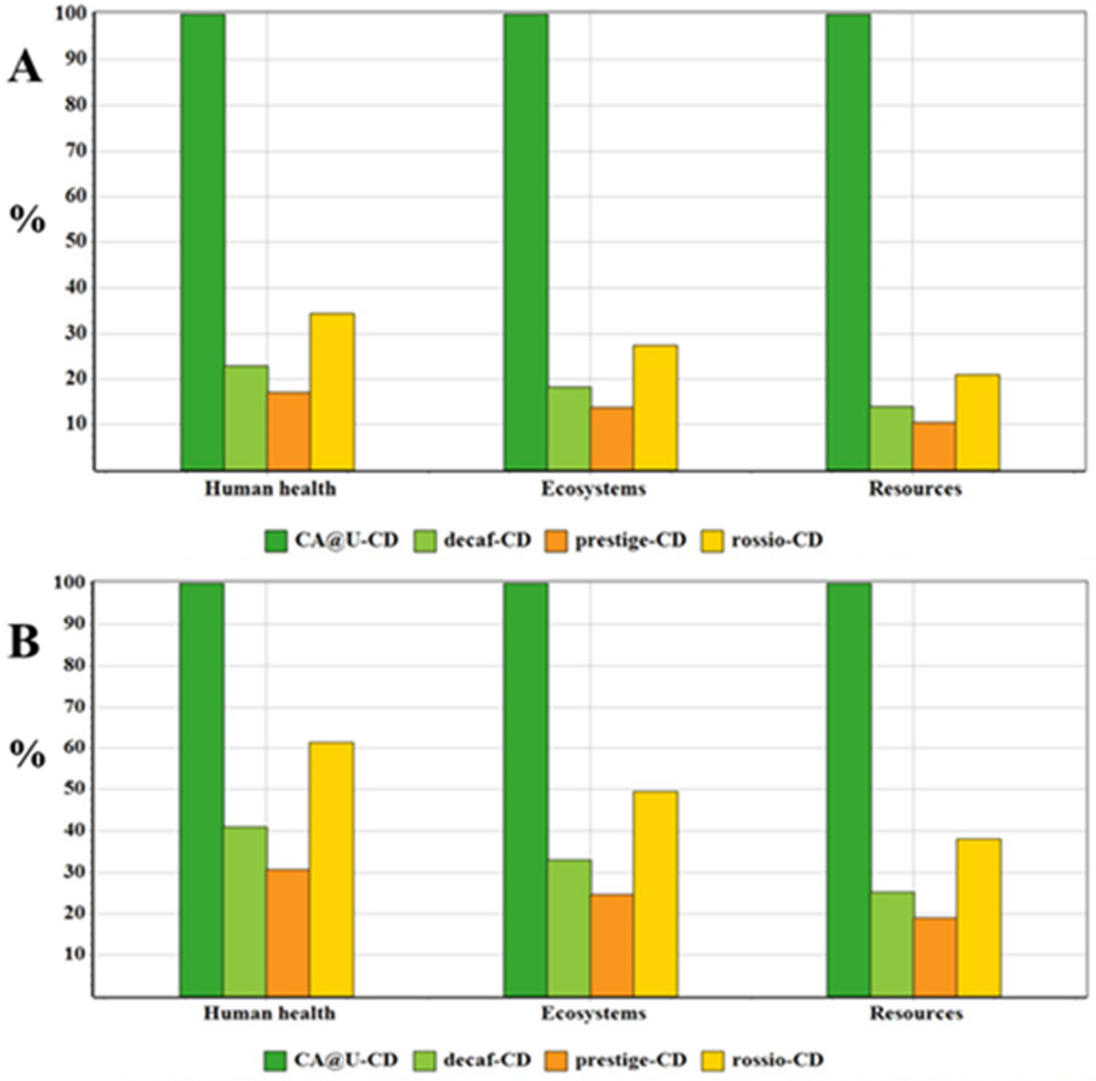

Figure S6. Comparative environmental profiles for the synthesis of all CDs, re-scaled with respect to the QY_{FL} of the CDs, by changing the input of electricity to 70% (A) and to 130% (B) of the original value.
